# How Does Internet Use Affect Mental Health of Rural Residents? The Mediating Role of the Neighborhood Social Environment

**DOI:** 10.3390/bs16060948

**Published:** 2026-06-09

**Authors:** Changxu Wang, Jinyong Guo

**Affiliations:** 1 College of Economics and Management, Huaibei Normal University, Huaibei 235020, China; wangcx@chnu.edu.cn; 2 College of Economics and Management, Jiangxi Agricultural University, Nanchang 330045, China

**Keywords:** internet use, neighborhood relationship, neighborhood trust, neighborhood identity, mental health

## Abstract

As digital technology has become increasingly integrated into rural governance and daily life in China, Internet use among rural residents exerts a multifaceted influence on their mental health. A key mechanism lies in its restructuring of the neighborhood social environment. Uncovering this mechanism is essential for understanding the theoretical and practical connections between rural social transformation and individual well-being in the digital age. This study applied a binary probit model to data from the 2020 China Family Panel Studies (CFPS) to examine the impact of Internet use on the mental health of rural residents. Mediation analysis was used to examine the role of the neighborhood social environment, and the conditional mixed process method was applied to address potential endogeneity issues. Empirical results demonstrate that access to the Internet, along with the breadth and depth of its use all significantly improve the mental health of rural residents. Internet use promotes mental health by strengthening neighborhood relationship and trust, whereas it also negatively affects mental health by suppressing neighborhood identity. Heterogeneity analyses reveal three key dimensions of variation. (1) By usage type: Activities such as gaming, short-video consumption, and WeChat communication show positive associations with mental health, whereas online shopping and learning exhibit non-significant effects. (2) By user group: The mental health benefits are more pronounced among women, less-educated individuals, and middle-aged to older adults. (3) By region: Positive associations are observed in central and western China, with the most substantial effect in the central region. This study elucidates the mechanism through which Internet use affects mental health: the restructuring of traditional, place-based social capital in rural neighborhoods. These findings offer robust empirical support for policies that integrate digital initiatives with the nurturing of local community bonds to improve rural mental health and foster livable and harmonious villages.

## 1. Introduction

Digital technologies are reshaping social relationship, bringing the Internet’s impact on the lifestyles and mental health of rural populations into sharp focus. As of January 2025, China’s rural Internet penetration rate stood at 69.2%, representing over 313 million users. This expanding connectivity is driving fundamental transformations in rural production and lifestyles (J. Hu et al., 2024). Consequently, a key question emerges: does Internet use alleviate or exacerbate psychological distress in rural residents?

The impact of Internet use on mental health remains a subject of ongoing debate in the academic literature. One line of research emphasizes a time–displacement effect, positing that online engagement may supplant face-to-face interactions, thereby diminishing social capital and negatively affecting mental health (X. Wang & Zhao, 2024; R. Zhang et al., 2024). Conversely, other scholars highlight an enhancement effect. They argue that the Internet, as a low-cost and efficient medium, transcends spatiotemporal barriers. Particularly for information-disadvantaged groups such as rural residents, it provides accessible channels that can amplify the mental health benefits derived from online learning, socialization, and entertainment (J. Hu et al., 2024; Marta, 2022; Wadman et al., 2025). Consequently, the central question persists: For rural residents, does Internet use function primarily as an empowering tool for mental well-being or as a potential psycho-social risk factor? This question warrants rigorous empirical examination.

Most current studies treat Internet use as a simple binary variable (Hou, 2025; Huoyun et al., 2023; Lam et al., 2020). However, with near-universal access, the critical differentiator of impact is no longer connectivity per se, but the nature and extent of usage. As noted by Chi et al, disparities in digital engagement can have more profound implications than disparities in basic access. Consequently, heterogeneity in digital engagement is likely a key driver of variation in mental health outcomes (Chi & Lin, 2026). Understanding this heterogeneity is thus crucial for designing digital interventions that genuinely empower and for crafting effective public health policies (Dong et al., 2025; Xiang & Xing, 2025). However, few studies have specifically examined how these different ways of using the Internet affect mental health.

A further critical question concerns how Internet use affects rural residents’ mental health. Existing research has predominantly focused on individual-level mechanisms, such as personal networks and social engagement (Kung & Steptoe, 2022; Sheng & Yujie, 2021; Tianyuan et al., 2023), while largely overlooking a pivotal community-level factor: the neighborhood social environment. As the primary social space of daily life, a supportive neighborhood environment is fundamental to psychological well-being (Kim et al., 2022; Leer et al., 2025; T. Lin et al., 2024). Therefore, centering the neighborhood social environment in our analysis provides a crucial theoretical bridge: it connects micro-level internet use behaviors to macro-level social transformation, thereby elucidating the unresolved “black box” of how Internet use influences the mental health of rural residents via the mediating channel of the community-level neighborhood social environment (Sui et al., 2022). By rigorously examining this mediating pathway, this study addresses the neglect of community-level mechanisms in the literature and offers a novel explanatory framework for understanding the social determinants of rural residents’ mental health in the digital era.

Based on this, our study expands the measurement of Internet use from simple access to include both breadth and depth of use. To advance beyond this binary paradigm, our study conceptualizes and measures Internet use along three distinct dimensions: access, breadth, and depth. This multidimensional approach allows for a more nuanced analysis of its heterogeneous associations with mental health outcomes (Wadman et al., 2025). Specifically, we first systematically assess how varying patterns of Internet use are differentially linked to the psychological well-being of rural residents. Second, we interrogate the underlying mechanism by focusing on the mediating role of the neighborhood social environment. Third, we conduct heterogeneity analyses to examine how these relationship vary across key regional and demographic subgroups.

This study makes three key contributions. First, it extends the conceptualization of Internet use beyond a binary measure of access to incorporate distinct dimensions of breadth and depth. Second, regarding mechanisms, we posit the “neighborhood social environment” as a pivotal community-level mediator. Consequently, the study provides a novel framework for understanding the digital restructuring of traditional rural social capital and its implications for individual well-being. Third, heterogeneity analysis reveals that the mental health benefits of Internet use are both inclusive and compensatory in nature, thereby revealing digital empowerment strategies can be leveraged to foster more equitable psychological well-being across social strata.

The remainder of this paper is structured as follows. Section 2 presents the theoretical analysis and research hypotheses, which form the theoretical framework of the study. Section 3 describes the data processing procedures and the empirical model used. Section 4 provides an analysis of the empirical results. The final section summarizes the study and presents the conclusions.

## 2. Theoretical Background and Research Hypothesis

### 2.1. The Concept of Neighborhood Social Environment

In the Chinese context, “neighborhood” constitutes a fundamental socio-spatial unit defined by both geographical proximity and sustained social interaction, often overlapping with the concept of “community”. It is academically defined as “a small-scale social group formed by residents in close proximity, characterized by a dense web of social interactions and a shared sense of identity” (Lei & Lin, 2021; Z. Liu et al., 2020). The neighborhood environment is broadly categorized into the built environment—encompassing physical structures, spaces, and amenities (Rao et al., 2007)—and the social environment, which refers to the network of relationships, norms, and resources generated through ongoing social exchange (Diez Roux & Mair, 2010). This study focuses explicitly on the neighborhood social environment. The neighborhood social environment encompasses the socio-ecological context within which individuals or groups access and exchange social resources through localized interactions (Subramanian et al., 2003). While its precise constitutive elements lack a universal definition in the literature, it is generally conceptualized as a multidimensional construct (Sampson et al., 1997). A consensus on the operational definition of the neighborhood social environment remains elusive, prompting geographers and sociologists to examine its constituents across varied spatial scales. Commonly examined dimensions include perceived safety, place identity, neighborly interactions, availability of emotional support, local social networks, and levels of community participation (Kepper et al., 2019; Yen & Syme, 1999).

For instance, Taking China urban community as the research background, Zhu and Fu proposed an eight-indicator framework across four dimensions: neighborhood support, cognition, interaction, and community participation (Zhu & Fu, 2017). Similarly, R. Wu et al. (2019), in their study of migrant populations in Guangzhou, employed proxies such as neighborhood harmony, migrant resident ratio, poverty level, and population density (R. Wu et al., 2019). W. Li et al. (2021), utilizing the 2016 CFPS data, measured this environment in old urban areas through neighborly relations, community belonging, and trust (W. Li et al., 2021). Notably, most extant operationalizations focus on urban settings, with rural contexts receiving comparatively scant attention. A foundational contribution is the work of M. Zhang et al. (2022), who measured the rural neighborhood social environment through informal interaction, trust, reciprocity, support, cohesion, and belonging (M. Zhang et al., 2022).

While a fully standardized measurement system for the neighborhood social environment remains elusive in the literature, three dimensions are consistently identified as its core constituents: neighborhood relationship, neighborhood trust, and neighborhood identity.

Neighborhood relationship denote the frequency and quality of social interactions among residents.

Neighborhood trust captures the mutual reliability and confidence shared between residents.

Neighborhood identity reflects an individual’s sense of belonging and emotional attachment to their local community.

Accordingly, this study adopts this established framework to deconstruct the rural neighborhood social environment. By operationalizing it through these three dimensions, we aim to rigorously analyze their specific mediating roles in the pathway linking Internet use to the mental health of rural residents.

### 2.2. The Impact of Internet Use on Mental Health Among Rural Residents

A substantial body of literature documents its significant positive effects, positing that the Internet can furnish often-scarce resources such as social support and a sense of belonging, thereby enhancing mental health (Y. Wang & Li, 2025; Xu et al., 2025). For example, existing research indicates that Internet use significantly promotes both physical and mental health among older adults in rural China (J. Hu et al., 2024). Similarly, research indicates that for rural left-behind women, online learning, socializing, and entertainment serve as vital pathways to improved mental health, with social and entertainment activities exerting the most pronounced impact (Mao et al., 2023).

However, some scholars argue that excessive Internet use increases dependency on digital media. This can lead to Internet addiction among “left-behind children”, posing risks to their mental health (Shang et al., 2024). Other scholars, drawing on the displacement theory, contend that increased Internet use reduces the time available for physical exercise and face-to-face social interaction among older adults. This reduction may subsequently increase feelings of depression and loneliness, harming their overall well-being (X. S. Chen et al., 2025; Du et al., 2023). Compared to urban areas, rural regions face more pronounced challenges, such as rural hollowing-out and family separation due to population outflow (Ma et al., 2024). In this context, the Internet plays a particularly important role for rural residents in maintaining social relationship and obtaining emotional support, even though its use may also involve certain negative effects. Based on the above, we propose the following hypothesis:

**H1.**  *The access, breadth, and depth of Internet use is associated with the mental health of rural residents*.

### 2.3. Analysis of Influence Mechanism

#### 2.3.1. The Impact of Internet Use on Neighborhood Relationship

The Internet is becoming deeply embedded in the daily lives and social practices of rural residents, profoundly reshaping their neighborhood social environment. However, academic findings on how Internet use influences neighborly relations remain inconsistent. X. Chen et al. (2023) found that the frequency of Internet use exerts a significant negative effect on the familiarity of neighborhood relationship—that is, higher usage correlates with weaker familiarity among neighbors (Wei & Jia, 2023).

In contrast, J. Zhou et al. (2023) offers a qualitative reinterpretation of rural sociality in the digital era (J. Zhou et al., 2023). He contends that despite the penetration of market forces and substantial village outmigration, the traditional acquaintance-based society has not necessarily eroded. Instead, rural residents leverage Internet technologies to preserve the core fabric of traditional rural society, thereby maintaining existing social ties. Crucially, social platforms like WeChat, which are anchored in pre-existing familiar relationship, transcend geographical constraints (X. Wu & Liu, 2025; Zhao et al., 2025). By creating a fluid, blended online–offline space—termed here as a “networked differential order pattern”—they cater to the diverse social needs of rural residents. This adaptive structure enhances the resilience and stickiness of neighborhood bonds, which in turn can positively influence residents’ mental health.

#### 2.3.2. The Impact of Internet Use on Neighborhood Trust

Neighborhood trust refers to the confidence that rural residents extend to fellow villagers, including those who have migrated for work. Drawing on information asymmetry theory from economics, the evaluation of such trust can be framed as a decision-making process wherein residents seek information to bridge knowledge gaps and form reliable judgments (Yan et al., 2022). In this context, the Internet functions as a mechanism for enhancing environmental transparency. Online platforms enable villagers to interact and exchange information regarding local affairs, thereby reducing information asymmetry and equalizing access to public discourse (Jin et al., 2017; H. Wang et al., 2009). This more transparent and participatory environment cultivates mutual trust, which is posited to be a critical social–psychological resource that enhances mental health (C. Y. Lin et al., 2021).

#### 2.3.3. The Impact of Internet Use on Neighborhood Identity

The Internet also functions as a pivotal medium for the construction of social identity, exerting a significant influence on rural residents’ sense of neighborhood belonging. Scholars note that in the digital age, the more open and liberalized flow of information enables internet use to facilitate downward social comparison among the middle class, thereby consolidating their class identity (Johnson, 2021). Conversely, for rural residents situated at the lower rungs of the socioeconomic hierarchy, increased exposure to information about social inequality online often prompts upward social comparison. This dynamic can foster a sense of relative deprivation, diminish their perceived social status, and ultimately impose adverse effects on their mental health (Park & Kim, 2024). Based on the above analysis, the following hypotheses are proposed:

**H2.**  *Internet use is associated with the mental health of rural residents through the neighborhood social environment*.

**H2a.**  *Internet use is positively associated with the mental health of rural residents by strengthening their neighborhood relationship*.

**H2b.**  *Internet use is positively associated with the mental health of rural residents by strengthening their neighborhood trust*.

**H2c.**  *Internet use is negatively associated with the mental health of rural residents by weakening their neighborhood identity*.

### 2.4. Due to the Digital Divide, Heterogeneity Exists in Internet Use Among Rural Residents

According to existing literature, findings on the relationship between Internet use and residents’ mental health remain inconsistent. This contradiction may stem from the diverse functions of the Internet, making it difficult for individual studies to capture all its impact dimensions (X. Wen & Tian, 2024). Most existing research measures rural residents’ Internet use through binary access or frequency of use, while rarely distinguishing the specific roles of different types of online activities.

**Figure 1:**
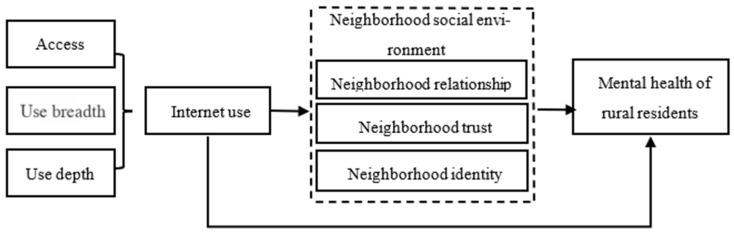
Theoretical framework.

With the advancement of digital rural initiatives, rural residents have largely bridged the first-level digital divide, achieving widespread network connectivity. However, disparities in the patterns and purposes of technology use can play a more decisive role in shaping societal outcomes than access alone (Y. Li et al., 2024; Oliver et al., 2024). Consequently, while improved infrastructure provides a baseline of equal connectivity, the resultant benefits are not uniformly distributed (Murphy et al., 2024). individuals possess varying digital capabilities, which influence the efficiency with which they can translate online engagement into tangible psychological gains (Kohli et al., 2024). Thus, it can be inferred that the Internet’s impact on mental health is not monolithic but is moderated by the purpose and manner of use, as well as by user demographics and regional contexts. Based on this analysis, the following hypothesis is proposed:

**H3.**  *Due to the digital divide, the impact of Internet use on the mental health of rural residents is heterogeneous. The theoretical framework of this study is presented in Figure 1*.

## 3. Data and Methodology

### 3.1. Data

The China Family Panel Studies (CFPS) is a nationwide, comprehensive, longitudinal social survey initiated by Peking University in 2010. It adopts a multi-stage probability sampling design with implicit stratification, covering 25 provinces, municipalities, and autonomous regions that represent approximately 95% of the Chinese population. Baseline data collection was conducted in 2010, with biennial follow-ups thereafter. The survey employs face-to-face computer-assisted personal interviewing (CAPI) to collect data at individual, household, and community levels, focusing on a wide range of domains including economic status, education, family dynamics, and health outcomes.

To investigate the relationship between Internet use, neighborhood social environment, and mental health among rural residents, this study utilizes data from the 2020 China Family Panel Studies (CFPS). The analytic sample was restricted to individuals officially registered as rural residents and currently residing in rural areas, following the standard demographic classification in China. After listwise deletion of missing values on key variables (Internet use, neighborhood social environment, mental health, and covariates), 199 cases were removed, yielding a final analytic sample of 8548 rural residents.

### 3.2. Variable Selection

#### 3.2.1. Dependent Variable

The dependent variable is the mental health status of rural residents, operationalized using the 8-item Center for Epidemiologic Studies Depression Scale (CES-D8), a widely validated instrument for assessing mental health (Han & Jia, 2012). Following the scale’s standard protocol, the two positively valenced items (“Feel happy” and “Enjoy life”) were reverse-coded. A continuous composite score (range: 4–32) was calculated by summing all items, with higher scores indicating more severe depressive symptoms and thus poorer mental health. Additionally, in line with the validated clinical cutoff, respondents with a total score of 16 or above were classified as having a depressive tendency (Radloff, 2015). This classification was used to create a binary variable (1 = depressive tendency; 0 = otherwise). The items used to assess mental health are detailed in Table 1.

#### 3.2.2. Explanatory Variables

The key explanatory variable is Internet use, which is conceptualized and measured along three distinct dimensions: access, breadth, and depth. This multidimensional approach allows for a nuanced analysis of how variations in digital engagement influence the mental health of rural residents (Du et al., 2023; H. Zhou et al., 2023).

Internet Access. This variable was operationalized through two survey items: “Do you use a computer to access the internet?” and “Do you use mobile devices such as smartphones to access the internet?” Respondents who answered affirmatively to either question were coded as 1 (users); all others were coded as 0 (non-users).

Breadth of Internet Use. The breadth of use was captured by inquiring about participation in five distinct online activities during the past week: online gaming, shopping, short-video consumption, online learning, and WeChat use. Each activity was dichotomously coded (1 = engaged; 0 = did not engage). A composite breadth score (range: 0–5) was generated by summing these codes, with higher scores indicating a wider repertoire of use.

Depth of Internet Use. Depth reflects the perceived centrality of the Internet in daily life, constructed from respondent perceived importance ratings. The CFPS survey solicited ratings on a 5-point Likert scale (1 = “very unimportant” to 5 = “very important”) for the importance of the Internet for five purposes: learning, work, social interaction, entertainment, and commercial activities. Following established methodology, a composite depth score (range: 5–25) was computed by summing the ratings across all five domains. Higher scores denote greater perceived importance and deeper embeddedness of the Internet in the user’s daily routine.

#### 3.2.3. Mediating Variable

Drawing on the rural Chinese context and prior literature, we measure the neighborhood social environment through three established dimensions: neighborhood relationship, trust, and identity (X. Chen et al., 2023; Huang et al., 2025).

Neighborhood relationship was assessed with the item: “Overall, how would you describe the relationship among neighbors in your village?” Original responses on a 5-point scale (1 = “very good” to 5 = “very poor”) were reverse-coded for intuitive interpretation (1 = “very poor”, 5 = “very good”).

Neighborhood trust was gauged by: “How much do you trust your neighbors?” Original responses on a 0–10 scale were converted to a standard 5-point scale (1 = “very distrustful”, 5 = “very trusting”).

Neighborhood identity was captured by: “What is your social status in this locality?” using a 5-point scale (1 = “very low”, 5 = “very high”).

#### 3.2.4. Control Variables

We control for variables at three levels:

Individual-Level: Gender, age, education, marital status, employment status, smoking, drinking, napping habits, chronic disease status, political affiliation, and income.

Household-Level: Social capital, household size, enrollment in pension insurance, and enrollment in cooperative medical insurance.

Village-Level: Regional dummies and the logarithmically transformed village economic development level.

#### 3.2.5. Descriptive Statistics

Descriptive statistics are presented in Table 2. The mean score for mental health (the dependent variable) is 0.329, indicating a moderate level of mental health in the sample. Regarding the explanatory variables, the mean for Internet access is 0.547, confirming that a majority (over half) of rural residents are Internet users. The mean breadth of Internet use is 1.492, suggesting a relatively narrow range of online activities. The mean depth score is 10.287, which reflects a moderate degree of importance attached to the Internet in daily routines.

For the mediating variables, neighborhood relationship show a high mean (4.072), denoting generally positive perceptions. Neighborhood trust has a mean of 3.649, pointing to a moderate but not strong level of trust. In contrast, neighborhood identity has the lowest mean among the three (3.189), indicating a comparatively weaker sense of social belonging within the local community.

### 3.3. Model Specification and Endogeneity Discussion

(1) Baseline Regression—Binary Probit Model

To estimate the effect of Internet use on the mental health of rural residents, we employ a binary probit model. This model is appropriate for the binary nature of the dependent variable (depressive tendency) and is estimated via maximum likelihood to ensure robust inference.

(1) $${Mental\_health}_{ij}=\alpha +\beta \;{\mathrm{int}ernet}_{ij}+X_{\mathrm{ij}}+\delta_{i}+\varepsilon_{ij}$$

In Equation (1), the dependent variable, ${Mental\_health}_{ij}$, represents the rural residents mental health status. It is a binary measure coded as 1 if the respondent exhibits a depressive symptoms and 0 otherwise. The core independent variable, ${\mathrm{int}ernet}_{ij}$, operationalizes Internet use through three distinct dimensions: access (a binary indicator), breadth, and depth. The $X_{ij}$ denotes a comprehensive set of control variables. The subscript j indexes individuals, $\delta_{i}$ represents province fixed effects, and $\varepsilon_{ij}$ is the idiosyncratic error term.

(2) Mediation Effect Test Model

To examine whether internet use affects mental health indirectly through the neighborhood social environment, we follow the procedure proposed by (Z. Wen & Ye, 2014) and specify the following models:

(2) $$M_{\mathrm{ijt}}=\alpha_{0}+\beta_{0}\mathrm{int}ernet_{ijt}+\gamma_{0}X_{ijt}+{\delta^{\prime \prime }}_{t}+{\theta^{\prime \prime }}_{j}+{\varepsilon^{\prime \prime }}_{ijt}$$

(3) $$Mental\_health_{\mathrm{ijt}}=\alpha_{1}+\beta_{1}internet_{ijt}+M_{ijt}+\gamma_{1}X_{ijt}+{\delta^{\prime }}_{t}+{\theta^{\prime }}_{j}+{\varepsilon^{\prime }}_{ijt}$$

In Equation (2), $M_{\mathrm{ijt}}$ denotes the mediator variable, which includes the neighborhood social environment. All other variables are the same as in Equation (1).

## 4. Empirical Results

### 4.1. The Impact of Internet Use on the Mental Health of Rural Residents

To clearly delineate the relationship between Internet use and mental health, we employ a hierarchical regression controlling for province fixed effects. We sequentially estimate the effects of the three core dimensions of Internet use—access, breadth, and depth—on mental health outcomes. The corresponding regression results are presented in Table 3.

1. The impact of Internet access on mental health.

Column (2) of Table 3 shows that Internet access maintains a marginally significant negative association with mental health problem (*p* < 0.10) after controlling for province fixed effects, corresponding to a 6.4% improvement in mental health. The stability of this coefficient confirms the robustness of the finding.

2. The impact of the breadth of Internet use on mental health.

Column (4) of Table 3 shows that a broader range of Internet use maintains a statistically significant negative association with mental health problem at the 5% level, even after accounting for province fixed effects. This indicates that engaging in a more diverse set of online activities—such as information seeking, entertainment, and commercial transactions—confers greater mental health benefits. In substantive terms, each additional type of online activity adopted is associated with a 2.6% reduction in the likelihood of depressive tendencies. The robustness of this finding is further confirmed by the stable coefficient across model specifications.

3. The impact of the depth of Internet use on mental health.

As shown in column (6) of Table 3, even after controlling for regional fixed effects, the depth of Internet use also shows a statistically significant negative association with mental health problems at the 5% level. This suggests that more intensive engagement in online activities—such as learning, entertainment, and commerce—is associated with a greater positive impact on mental health. Specifically, a one-unit increase in the depth of Internet use is associated with a 1.0% reduction in mental health problems among rural residents. The consistency in the sign and significance of the coefficient before and after including control variables confirms the robustness of this result.

### 4.2. Mechanism Analysis

The results presented in columns (2), (4), and (6) of Table 4 indicate that Internet use exerts statistically significant and differential effects on all three dimensions of the neighborhood social environment. Specifically, Internet use positively predicts stronger neighborhood relationship and greater neighborhood trust, yet it negatively predicts the level of neighborhood identity.

The mediation analysis quantifies the distinct contributions of each pathway. The beneficial effect is primarily channeled through neighborhood trust, which mediates 46.72% of the total effect. In comparison, the mediating role of neighborhood relations is notably smaller, accounting for only 8.30%. Conversely, the adverse pathway via the diminishment of neighborhood identity is predominant, mediating 56.72% of the total effect. Taken together, these estimates reveal an asymmetric dual mechanism: the negative impact of eroded neighborhood identity outweighs the combined positive buffering effects of neighborhood trust and relations, underscoring the dominant role of identity-based pathways in shaping mental health outcomes.

Consequently, Internet use influences the psychological well-being of rural residents through two countervailing mechanisms of unequal strength: a beneficial effect largely mediated by neighborhood trust, and a stronger, adverse effect mediated by the erosion of neighborhood identity. These results provide robust empirical support for the dual mediating mechanisms posited in Hypothesis H2.

### 4.3. Robustness Check: Alternative Measures of Depression

To assess the robustness of our baseline findings, we conducted an auxiliary analysis using an alternative operationalization of the dependent variable. Specifically, we replaced the 8-item Center for Epidemiologic Studies Depression Scale (CES-D8) with its full 20-item version (CES-D20), available in the CFPS 2020 survey. Both are summed continuous scores (CES-D8: 8–32; CES-D20: 20–80), which we analyzed using ordinary least squares (OLS) regression.

As presented in Table 5, the results of these robustness checks are fully consistent with our main findings. Internet access, breadth of use, and depth of use remain statistically significant predictors of depressive symptoms measured by both the CES-D8 and CES-D20 scales. Crucially, the direction and significance of all key coefficients are unchanged. This convergence of evidence across distinct measurement instruments strongly confirms the robustness of our core results.

### 4.4. Endogeneity

To address potential endogeneity bias, this study employed the Conditional Mixed Process (CMP) method for re-estimation (Roodman, 2011). Following the approach of (Agarwal et al., 2005), we used “the average Internet usage rate among other households in the individual’s village” as the instrumental variable.

The rationale for this choice is that within the same village, there are behavioral interactions and shared infrastructure effects, making this variable correlated with an individual’s own Internet use. At the same time, it is theoretically unrelated to the random error term in the model. The CMP results in Table 6 show that the instrumental variable is statistically significant at the 1% level in the first-stage regression, meeting the relevance requirement. The significant coefficient for atanhrho_12 confirms the presence of endogeneity. After controlling for endogeneity, the positive effect of Internet use remains statistically significant. This finding strengthens the robustness of our main conclusions.

### 4.5. Heterogeneity Effects

#### 4.5.1. Heterogeneity by Type of Internet Use

As Internet adoption becomes near-universal in rural China, analyzing mere access is insufficient to understand its heterogeneous impacts on mental health. This study therefore examines how different purposes of Internet use are associated with mental health outcomes. The regression results are presented in Table 7. The analysis reveals that using the Internet for online gaming, short-video consumption, and WeChat is significantly associated with better mental health. These activities likely function as accessible forms of leisure and social connection, providing entertainment and stress relief that contribute positively to mental health. Notably, the use of WeChat exhibits the strongest estimated protective effect (coefficient = −0.302), suggesting it plays a particularly substantial role in improving mental health among rural residents.

In contrast, the estimated coefficients for online shopping and online learning are positive but not statistically significant, suggesting that these activities are not associated with a discernible improvement in mental health at conventional levels. Online shopping could introduce psychological costs related to transactional friction, delivery uncertainties, or concerns about product quality, particularly for users with lower digital literacy. Similarly, online learning may have lower cultural acceptance or perceived relevance among certain rural demographics, especially older adults who may be less familiar with or skeptical of formal digital education platforms.

In summary, our analysis reveals a protective association between specific leisure-oriented Internet uses—online gaming, short-video consumption, and WeChat communication—and the mental health of rural residents, as they are linked to a significant improvement in mental health. In contrast, the associations for online shopping and online learning are not statistically significant and thus do not support a clear beneficial effect within our sample.

#### 4.5.2. Heterogeneity Analysis by Gender, Education, and Age

To assess how the relationship between Internet use and mental health varies across population subgroups, we conducted stratified analyses by gender, education level (dichotomized as lower/higher), and age cohort (middle-aged & older adults vs. younger adults). The results, presented in Table 8, reveal significant heterogeneity.

Gender. The protective association between Internet use and mental health is statistically significant for female rural residents but not for their male counterparts, although the direction of the coefficient is consistent. This differential effect may be attributed to variations in information processing and digital engagement, where female residents might be more responsive to the socio-emotional content and support accessible through these platforms.

Education. A pronounced differential effect is observed by education level. Internet use exhibits a significant protective association for residents with lower education, with a larger effect size compared to the higher-education group, for whom the association is positive but not significant. This pattern may reflect the information channel effect: for the lower-education group, the Internet primarily functions as a tool for bridging information and skill gaps, thereby enhancing psychological well-being. Conversely, individuals with higher education, while also accessing more information, may encounter a broader range of reference groups online, which could trigger upward social comparison and associated anxiety, potentially attenuating the net mental health benefit.

Age. The analysis reveals a distinct gradient in the mental health returns to Internet use across age cohorts. The protective association is most pronounced and statistically significant among middle-aged and elderly population (45 years and above). In contrast, for the younger cohort (below 45 years), the association is positive in direction but not statistically significant, suggesting a substantially weaker or more variable effect within this group. This pattern aligns with a digital compensation perspective. For younger rural residents, digital technologies are often ambient and deeply integrated into the social fabric, potentially offering diminishing marginal psychological utility as a novel resource. For middle-aged and older adults, however, Internet access may serve as a critical compensatory tool, effectively bridging gaps in social connectivity, information access, and leisure opportunities that are more constrained in their offline environments. The relatively larger effect size observed in this group underscores the high marginal value of digital inclusion for populations traditionally on the disadvantaged side of the digital divide.

#### 4.5.3. Regional Heterogeneity Analysis

To examine regional heterogeneity, we stratified the sample into eastern, central, and western sub-samples based on the standard classification of the National Bureau of Statistics and estimated the models separately for each region.

As presented in Table 9, the association between Internet use and mental health exhibits marked regional variation. The effect is statistically significant and strongest in magnitude in central China, remains significant but is attenuated in the western region, and is not statistically significant in the more developed eastern region.

## 5. Discussion and Conclusions

The creation of “healthy environments” constitutes a pivotal pathway within the “Healthy China” national strategy, which adopts a holistic definition of health—not merely the absence of disease, but a state of complete physical, mental, and social well-being. In rural China, the neighborhood social environment—the primary socio-spatial arena for daily life and interaction—is of fundamental importance. Cultivating a supportive milieu within this environment is critical for fostering the mental health of rural residents.

Currently, the Chinese push for digital village development is actively transforming these traditional social landscapes. Internet technologies are dissolving the historical geographical constraints of rural communities, migrating kinship- and locality-based ties—the bedrock of the traditional “acquaintance society”—onto digital platforms (X. Wu & Liu, 2025). This migration expands the horizons of neighborhood interaction, reinforces community bonds, and fundamentally reshapes the very fabric of the neighborhood social environment that underpins daily life and economic activities. This restructuring, in turn, exerts a profound influence on residents’ psychological states and behavioral patterns (Wan et al., 2025).

Consequently, a pressing scholarly and policy question emerges: how can the Internet be leveraged to construct a perceivable and accessible neighborhood social environment, thereby enhancing the mental health of rural residents? To address this, our study examines rural Internet use through a three-dimensional lens—access, breadth, and depth—and measures the neighborhood social environment at the village level. We empirically assess the differential impacts of various Internet use behaviors on mental health, systematically elucidate the mediating mechanisms through distinct dimensions of the social environment, and uncover the heterogeneous effects across different demographic groups and geographical regions.

First, our study establishes a robust positive association between multidimensional Internet engagement and the psychological well-being of rural residents. The empirical evidence confirms that access to the Internet, a broader scope of online activities, and deeper integration of digital tools into daily life are each independently associated with a significant reduction in depressive symptom. Regarding internet access, connectivity itself serves as a foundational protective factor. Rural residents with Internet access report consistently lower levels of depressive symptoms, supporting the premise that basic digital inclusion can act as a buffer against mental health risks in geographically and socially constrained settings (Z. Y. Li, 2025). Consistent with this view, scholars have provided macro-level validation for this, showing that initiatives like China’s Digital Village Construction are associated with reduced depression among rural adults, with particularly strong benefits for disadvantaged groups (Wan et al., 2025). Furthermore, Cross-national studies suggest that such non-linear patterns may generalise beyond China: a recent meta-analysis of 223 studies found that Internet use is moderately associated with depressive symptoms and subjective well-being (Cai et al., 2023). This evidence solidifies internet access as a foundational protective factor, buffering mental health risks in constrained settings.

Regarding the breadth of use, we find that digital diversification confers mental health benefits. Engagement across a spectrum of online domains—encompassing informational (e.g., learning), recreational (e.g., entertainment), and transactional (e.g., commerce) activities—is linked to markedly lower depression scores. This suggests that the Internet’s value lies not in any single function but in its capacity to support a heterogeneous portfolio of needs, thereby enriching the user’s psychosocial resources and resilience (C. L. Li et al., 2025). This finding aligns with the concept of “combinatorial digital benefits” in recent digital health research (Bond et al., 2023). For instance, some scholars propose that using multiple digital applications with different functions (referred to as a “poly-digital” mode) can lead to cumulative health gains by fulfilling diverse psychosocial needs (Löchner et al., 2025; Taylor et al., 2020; Zeng et al., 2025). This evidences collectively indicates that the core value of the Internet lies in its capacity to serve as an integrated “toolbox,” rather than relying on any single function.

Finally, concerning the depth of use, the perceived importance and embeddedness of the Internet in daily routines emerged as a critical predictor. Deeper, more integral use of online platforms and functions is associated with greater alleviation of psychological distress. This finding underscores a qualitative shift in digital engagement: beyond mere access or breadth, it is when online tools become deeply embedded and indispensable for managing daily life, fostering social connections, and shaping self-concept that they most effectively build psychological resilience and mitigate the risk of mental disorders. However, the existing literature, including studies like the one by Bhatiasevi (2024), often focuses on specific platforms (e.g., social media) or general usage patterns (Bhatiasevi, 2024). Our study advances this discourse by conceptualizing and empirically validating “depth of use” as a distinct and qualitative dimension of digital engagement.

Second, our mechanism analysis delineates that the impact of Internet use on mental health is not merely direct but is significantly mediated by a restructuring of the neighborhood social environment. Internet use influences psychological well-being through a complex socio-psychological pathway, primarily by differentially shaping its three core dimensions: neighborhood relationship, trust, and identity.

Specifically, Internet use promotes mental health by strengthening neighborhood relationship and trust. As posited by social capital theory: the Internet functions as a critical infrastructure for social engagement. It expands the scale and frequency of social participation for rural residents (Guo et al., 2022; Q. Liu et al., 2020), facilitates resource exchange, and fosters the development of denser local networks. This digitally augmented interaction reinforces relational bonds and cultivates reliability among neighbors, providing a profound sense of emotional belonging (M. Chen & Li, 2024). Consequently, this enriched social fabric acts as a buffer against psychological distress, effectively mitigating feelings of loneliness, depression, and social alienation.

Conversely, the test also reveals a countervailing mechanism: while internet access shows a weak positive correlation with neighborhood relationship (β = 0.047, *p* < 0.1), it significantly erodes neighborhood identity (β = −0.321, *p* < 0.001), which in turn exacerbates mental health distress (β = −0.113, *p* < 0.001). The digital era has fundamentally altered the sources of this perception. The Internet, and social media in particular, has become a dominant force in shaping class and status awareness. However, persistent regional and socioeconomic disparities in China place many rural residents at a structural disadvantage (P. Wen et al., 2022; H. Zhang et al., 2025). According to social comparison theory, exposure to the curated lives and achievements of broader, often idealized online “reference groups” through platforms like WeChat can readily trigger upward social comparisons. This process frequently induces a sense of relative deprivation as individuals benchmark themselves against these distant, elevated standards, thereby diminishing their satisfaction with and identification with their local social position (neighborhood identity) (W. Wang et al., 2020). This attenuated local identity, in turn, constitutes a significant psychosocial risk factor that negatively impacts mental health. Chen’s research supports our framework by revealing how internet use expands individuals’ frames of social reference (F. Chen et al., 2025). This explains the altered comparative context for rural residents. Our contribution specifically identifies the psychological pathway where exposure to curated online lives triggers upward social comparison with distant reference groups, thereby eroding local identity and elevating mental health risks.

Third, heterogeneity analyses uncover two defining features of the Internet’s mental health effects in rural China: inclusivity and a compensatory logic. The benefits are not uniformly distributed but are systematically more pronounced among groups and in regions where traditional resources are scarcest.

Regarding usage purposes, engagement in leisure and communication—such as online gaming, short-video consumption, and WeChat use—is consistently associated with better mental health. In contrast, more transactional or formal activities like online shopping and online learning show null effects. Specifically, for online learning, its limited penetration and psychological impact in rural areas can be traced to: (1) High access thresholds: Requirements for sustained attention, digital literacy, and self-directed learning may exceed the capacity or habits of many residents (Prosen & Ličen, 2024); (2) Lack of a supportive ecosystem: The absence of formal accreditation, peer support, or tutoring mechanisms common in urban e-learning environments undermines completion rates and satisfaction (Safdar et al., 2022). For online shopping, the null effect may stem from: (1) Transactional friction: Concerns over payment security, product authenticity, and complex return policies can generate anxiety rather than convenience (Senali et al., 2024; Sun et al., 2023); (2) Logistical constraints: Underdeveloped delivery networks in remote villages increase costs and waiting times, negating core advantages (Yulia et al., 2022); (3) Preference for tangible trust: In communities where commerce is traditionally based on face-to-face interaction and reputation, impersonal online transactions may not adequately fulfill the social trust component integral to positive consumption experiences (Costanza et al., 2022). This pattern suggests that the Internet’s primary psychological value in this context stems from fulfilling core psychosocial needs for relaxation, leisure, and connection, rather than from its instrumental utility for commerce or formal education.

Regarding user groups, the compensatory nature of the effect is stark. The mental health returns are significantly concentrated among women, less-educated individuals, and middle-aged to older adults—groups that often face greater social constraints or have fewer alternative resources. For them, the Internet appears to function as a levelling mechanism, granting access to social participation, informational resources, and entertainment that can offset offline disadvantages. Conversely, for groups like men, the highly educated, and younger adults—who typically possess more robust pre-existing social and economic capital—the marginal psychological benefit of additional Internet use is attenuated or non-significant (Snodgrass et al., 2018). The study by Zhang et al. demonstrates the positive link between Internet use and resource-deprived older adults’ well-being (J. W. Hu & Tan, 2025). Our research advances this by identifying that this benefit is a specific compensatory effect, where the Internet acts as a crucial leveling mechanism for underserved groups by fulfilling their unmet psychosocial needs.

Finally, regarding regional heterogeneity, the effect follows a clear developmental gradient. It is strongest in central China, remains significant but weaker in the west, and is not significant in the east. This pattern aligns with an inverted-U relationship between regional digital maturity and well-being returns. In the eastern region, where digital infrastructure and adoption are mature, the marginal psychological benefit of additional Internet use may have plateaued. Moreover, International evidence suggests that the psychological returns to internet access follow a pattern of diminishing marginal returns after certain penetration thresholds are reached (Orben et al., 2022; Cai et al., 2023). Conversely, in the western region, which is in an earlier phase of digital diffusion, the full suite of potential benefits may not yet be fully realized (Jing & Qing, 2023). The central region appears to occupy an optimal intermediate position—having assimilated digital technologies sufficiently to yield substantial benefits, yet not so thoroughly that returns have diminished—resulting in the most pronounced observed effect.

## 6. Policy Recommendations

1. Actively cultivate Internet use patterns that enhance psychological well-being, rather than focusing solely on access.

Central and local governments should ensure that policies must transcend basic infrastructure provision by guiding and training rural residents—especially those with low digital literacy—to use the Internet for diversified leisure, meaningful social connection, and substantive learning, thereby maximizing its mental health dividends.

2. Harness online platforms to strengthen place-based social capital while proactively mitigating the risks of adverse social comparison.

Community organizations (e.g., village committees) can support the creation of village-level digital communities (e.g., WeChat groups) to reinforce offline ties and mutual support. In parallel, integrate critical digital literacy education into community programs to help residents, particularly vulnerable groups, healthily process online information and build resilience against the negative psychological effects of upward social comparison.

3. Prioritize targeted, compensatory digital interventions for subgroups that demonstrate the greatest well-being returns.

Local governments and non-governmental organizations (NGOs) direct resources and tailored programs toward traditionally underserved groups (e.g., women, the less-educated, older adults). This includes developing accessible training materials, providing device subsidies, and co-designing locally relevant online services to ensure digital dividends are distributed equitably and inclusively.

## 7. Limitations and Future Research Directions

This study is subject to several limitations.

First, despite our efforts to include a comprehensive set of control variables to mitigate omitted variable bias, the possibility of residual confounding cannot be entirely ruled out. Furthermore, we used listwise deletion to handle missing data, which may lead to reduced statistical power, significant information loss, and potential sample bias if the missingness is not completely at random.

Second, the operationalization of the mediating mechanisms warrants further refinement. While we establish the neighborhood social environment as a significant mediator, our measures of relationship, trust, and identity may not exhaustively capture these multifaceted constructs. Moreover, a systematic assessment of the neighborhood built environment—a dimension prominent in urban studies but less examined in rural contexts—is absent. Key features such as land-use patterns, population density, and transportation infrastructure in villages were not incorporated, limiting a holistic understanding of the “neighborhood environment” mechanism.

Third, the representativeness of our sample and the generalizability of the findings merit further scrutiny. Although based on a national survey, the data may not adequately capture the distinct social fabric and digital trajectories of specific village typologies (e.g., “hollowed-out” villages, urban-fringe villages). Consequently, the transferability of our conclusions across diverse rural settings requires validation through more context-specific investigations.

To address these gaps, we propose three avenues for future inquiry.

Refining Causal Identification. Future work should seek to incorporate more precise or novel instrumental variables, leverage longitudinal or quasi-experimental designs, and integrate richer datasets to better isolate the causal effect of Internet use on mental health.

Deepening and Integrating Mechanistic Pathways. Employing mixed-methods approaches is crucial to unpack the nuanced processes, particularly the socio-psychological underpinnings of diminished neighborhood identity. Furthermore, developing an integrated theoretical framework that encompasses both the social and built dimensions of neighborhood environments will provide a more complete test of the mediating pathways through which digital connectivity influences mental health.

Expanding contexts and embracing interdisciplinary lenses. Research should explore the psychological implications of emerging digital formats (e.g., AI-driven content, short videos) and compare effects across heterogeneous rural development models. We also encourage cross-disciplinary collaboration.

## Figures and Tables

**Table 1:** Measurement of Mental Health.

Variable	Measurement	Survey Items	Response Options
Mental Health	CES-D8	1. I felt depressed during the past week. 2. I felt that everything I did was an effort during the past week. 3. My sleep was restless during the past week. 4. I was happy during the past week. 5. I felt lonely during the past week. 6. I enjoyed life during the past week. 7. I felt sad during the past week. 8. I felt that I could not get going during the past week.	1 = Rarely or none of the time (<1 day) 2 = Some or a little of the time (1–2 days) 3 = Occasionally or a moderate amount of the time (3–4 days) 4 = Most or all of the time (5–7 days)

**Table 2:** Variable Description.

Variable	Variable Definition	Mean	Standard Deviation	Min	Max
Dependent Variable					
Mental Health	1 = Presence of depression, 0 = Absence of depression	0.329	0.47	0	1
Explanatory Variables					
Internet Access	0 = Non-internet user, 1 = Internet user	0.547	0.498	0	1
Breadth of Internet Use	Coverage of internet usage modalities, coded 0–5	1.492	1.555	0	5
Depth of Internet Use	Summed score based on perceived importance of internet usage, range 5–25	10.287	9.88	0	25
Mediating Variables					
Neighborhood Relationship	Based on actual rating score (1–5 scale)	4.072	0.863	1	5
Neighborhood Trust		3.649	0.846	1	5
Neighborhood Identity		3.189	1.096	1	5
Control Variables					
Gender	0 = Female, 1 = Male	0.495	0.5	0	1
Age	Actual age	47.875	50.346	18	85
Educational Attainment	0 = Illiterate	1.537	1.191	0	4
	1 = Primary school				
	2 = Junior high school				
	3 = Senior high school				
	4 = College or above				
Marital Status	1 = Married, 0 = Unmarried	0.801	0.399	0	1
Employment Status	1 = Employed, 0 = Unemployed	0.398	1.948	0	1
Political Affiliation	1 = Communist Party member, 0 = Non-member	0.011	0.106	0	1
Smoking Status	1 = Smoker, 0 = Non-smoker	0.294	0.456	0	1
Alcohol Consumption	1 = Alcohol drinker, 0 = Non-drinker	0.124	0.329	0	1
Midday Nap Habit	1 = Takes midday nap, 0 = Does not take midday nap	0.597	0.491	0	1
Chronic Disease Status	1 = Has chronic disease, 0 = No chronic disease	0.148	0.355	0	1
Household Income	unit: CNY	18,314.362	20,024.844	0	344,000
Social Capital	Household expenditure on gifts and social etiquette (unit: CNY)	3488.731	5677.517	0	120,000
Household Size	Number of household members	4.57	2.089	1	15
Medical Insurance Coverage	1 = Covered by medical insurance, 0 = Not covered	0.496	0.5	0	1
Pension Insurance Coverage	1 = Covered by pension insurance, 0 = Not covered	0.894	0.308	0	1
Village Economic Development Level	Per capita income of village	17,873.224	9904.856	0	263,176
Central Region Dummy	Regional dummy variable (1 = Central region, 0 = Otherwise)	0.293	0.455	0	1
Western Region Dummy	Regional dummy variable (1 = Western region, 0 = Otherwise)	0.356	0.479	0	1

**Table 3:** Benchmark Regression Results.

Variable	(1)	(2)	(3)	(4)	(5)	(6)
Internet Access	−0.262 ***	−0.064 *				
	(0.03)	(0.03)				
Breadth of Internet Use			−0.094 ***	−0.026 **		
			(0.01)	(0.01)		
Depth of Internet Use					−0.017 ***	−0.010 **
					(0.00)	(0.00)
Gender		−0.204 ***		−0.205 ***		−0.187 ***
		(0.04)		(0.04)		(0.05)
Age		−0.001 *		−0.001 *		−0.001
		(0.00)		(0.00)		(0.00)
Educational Attainment		−0.085 ***		−0.081 ***		−0.090 ***
		(0.02)		(0.02)		(0.02)
Marital Status		−0.217 ***		−0.223 ***		−0.198 ***
		(0.04)		(0.04)		(0.06)
Employment Status		0.071 ***		0.069 ***		0.073 ***
		(0.01)		(0.01)		(0.01)
Political Affiliation		−0.199		−0.194		−0.191
		(0.15)		(0.15)		(0.16)
Smoking Status		0.072 *		0.073 *		0.073
		(0.04)		(0.04)		(0.06)
Alcohol Consumption		0.043		0.043		0.137 **
		(0.05)		(0.05)		(0.07)
Midday Nap Habit		−0.097 ***		−0.096 ***		−0.063
		(0.03)		(0.03)		(0.04)
Chronic Disease Status		0.440 ***		0.439 ***		0.458 ***
		(0.04)		(0.04)		(0.07)
Household Income		−0.077 ***		−0.077 ***		−0.096 ***
		(0.02)		(0.02)		(0.03)
Social Capital		0.003		0.003		0.009
		(0.01)		(0.01)		(0.01)
Household Size		−0.011		−0.010		−0.006
		(0.01)		(0.01)		(0.01)
Medical Insurance Coverage		−0.004		−0.004		−0.032
		(0.03)		(0.03)		(0.04)
Pension Insurance Coverage		−0.188 ***		−0.189 ***		−0.179 ***
		(0.05)		(0.05)		(0.06)
Village Economic Development Level		−0.110 ***		−0.108 ***		−0.012
		(0.04)		(0.04)		(0.05)
Central Region Dummy		0.043		0.043		0.065
		(0.04)		(0.04)		(0.05)
Western Region Dummy		0.160 ***		0.161 ***		0.174 ***
		(0.04)		(0.04)		(0.05)
cons	−0.302 ***	1.868 ***	−0.306 ***	1.847 ***	−0.251 ***	1.128 **
	(0.02)	(0.35)	(0.02)	(0.35)	(0.09)	(0.46)
*N*	8548	8548	8548	8548	4679	4679

Note: *** *p* < 0.01, ** *p* < 0.05, * *p* < 0.10. Standard errors are reported in parentheses.

**Table 4:** Mechanism Testing Results.

Variable	Mental Health	Neighborhood Relationship	Mental Health	Neighborhood Trust	Mental Health	Neighborhood Identity	Mental Health
	(1)	(2)	(3)	(4)	(5)	(6)	(7)
Internet access	−0.064 *	0.047 *	−0.060 *	0.180 **	−0.060 *	−0.321 ***	−0.101 ***
	(0.03)	(0.03)	(0.03)	(0.11)	(0.04)	(0.03)	(0.03)
Neighborhood relationship			−0.113 ***				
			(0.02)				
Neighborhood trust					−0.166 ***		
					(0.01)		
Neighborhood identity							−0.113 ***
							(0.01)
cons	1.868 ***		2.339 ***	0.855	2.915 ***		2.241 ***
	(0.35)		(0.36)	(1.11)	(0.37)		(0.36)
*N*	8548	8548	8548	8548	8548	8548	8548

Note: *** *p* < 0.01, ** *p* < 0.05, * *p* < 0.10. Standard errors are reported in parentheses; Mediation effect estimates = indirect effect/total effect × 100%.

**Table 5:** Robustness Testing.

Variable	CESD8			CESD20		
Internet Access	−0.302 ***			−0.602 ***		
	(0.11)			(0.21)		
Breadth of Internet Use		−0.102 ***			−0.203 ***	
		(0.04)			(0.07)	
Depth of Internet Use			−0.027 **			−0.046 *
			(0.01)			(0.03)
Control Variables	Control	Control	Control	Control	Control	Control
regional dummy variable	Control	Control	Control	Control	Control	Control
*R* ^2^	0.081	0.081	0.049	0.081	0.081	0.035
*N*	8548	8548	4679	8548	8548	4679

Note: *** *p* < 0.01, ** *p* < 0.05, * *p* < 0.10.

**Table 6:** Endogeneity Test.

Variable	First-Stage (1)	(2)	Second-Stage (3)
Internet Access			−0.036 *
			(0.04)
average Internet use rate	0.599 ***		
	(0.10)		
Control Variables	Control	Control	Control
regional dummy variable	Control	Control	Control
atanhrho_12		−0.294 ***	
		(0.077)	
*N*		8548	

Note: *** *p* < 0.01, * *p* < 0.10. Standard errors are reported in parentheses.

**Table 7:** Regression Results of Internet Use Purpose.

Variable	(1)	(2)	(3)	(4)	(5)
Online gaming	−0.119 **				
	(0.05)				
Online shopping		0.056			
		(0.04)			
Short-video consumption			−0.281 *		
			(0.15)		
Online learning				0.008	
				(0.06)	
WeChat					−0.302 **
					(0.12)
Control Variables	Control	Control	Control	Control	Control
Regional dummy variable	Control	Control	Control	Control	Control
*N*	4679	4679	4679	4679	4679

Note: ** *p* < 0.05, * *p* < 0.10. Standard errors are reported in parentheses.

**Table 8:** Regression Results of Individual Heterogeneity.

Variable	(1) Male	(2) Female	(3) Lower-Education Group	(4) Higher-Education Group	(5) Middle-Aged and Elderly Population	(6) Youth Group
Internet Access	−0.071	−0.092 *	−0.075 **	0.038	−0.077 *	−0.067
	(0.05)	(0.05)	(0.04)	(0.12)	(0.05)	(0.07)
Control Variables	Control	Control	Control	Control	Control	Control
Regional dummy variable	Control	Control	Control	Control	Control	Control
*N*	4234	4314	6786	1762	4849	3699

Note: ** *p* < 0.05, * *p* < 0.10. Standard errors are reported in parentheses.

**Table 9:** Results of Regional Heterogeneity Regression.

Variable	Eastern Region of China	Middle Part of China	Western Region of China
Internet Access	−0.317	−0.417 *	−0.320 *
	(0.20)	(0.22)	(0.18)
Control Variables	Control	Control	Control
*N*	2927	2502	3045

Note: * *p* < 0.10. Standard errors are reported in parentheses.

## Data Availability

The datasets generated and analyzed during the current study were derived from the China Family Panel Studies (CFPS). They are opened to everyone. Researchers who want to use these data can visit https://www.isss.pku.edu.cn/cfps/ (accessed on 11 May 2025).
